# Commercially Available Mobile Apps With Family Behavioral Goal Setting and Tracking for Parents: Review and Quality Evaluation

**DOI:** 10.2196/41779

**Published:** 2023-10-13

**Authors:** Summer Joy Weber, Shelagh A Mulvaney, Anthony Faiola, Madeline Brown, Tatsuki Koyama, Lili Sun, Susanna Lynn Goggans, Pamela Carmen Hull

**Affiliations:** 1 Markey Cancer Center University of Kentucky Lexington, KY United States; 2 School of Nursing Vanderbilt University Nashville, TN United States; 3 Department of Biomedical Informatics Vanderbilt University Medical Center Nashville, TN United States; 4 Department of Health and Clinical Sciences University of Kentucky Lexington, KY United States; 5 Department of Biostatistics Vanderbilt University Medical Center Nashville, TN United States; 6 College of Medicine University of Kentucky Lexington, KY United States; 7 Department of Behavioral Science College of Medicine University of Kentucky Lexington, KY United States

**Keywords:** goal setting, goal tracking, nutrition, health behavior, nutrition, parents, children, mobile apps

## Abstract

**Background:**

Goal setting and tracking are well established behavior change techniques. Little is known about the extent to which commercially available mobile apps are designed to guide parents in using these strategies, their evidence base, and their quality.

**Objective:**

This study aims to review commercially available apps that target parents in relation to setting and tracking behavioral goals for their children. The objectives were to classify the apps’ general characteristics, features, evidence base, and target behaviors and assess app quality overall and separately for apps that target health-related behaviors (HRBs) and apps without a health-related behavior (WHRB).

**Methods:**

Apps were identified using keyword searches in the Apple App Store and Google Play in the United States. Apps were included if their primary purpose was to assist with setting goals, tracking goals, tracking behaviors, or giving feedback pertaining to goals for children by parents. App characteristics and common features were documented and summarized. Two reviewers assessed app quality using the Mobile App Rating Scale (MARS). Descriptive statistics summarized the MARS total score, 4 quality subscales, and 6 app-specific items that reflect the perceived impact of the app on goal setting and tracking, overall and with subgroup analysis for HRB and WHRB apps.

**Results:**

Of the 21 apps identified, 16 (76%) met the review criteria. Overall, 9 apps defined and targeted the following HRBs: nutrition and mealtime (6/16, 38%), physical activity and screen time (5/16, 31%), sleep (7/16, 44%), and personal hygiene (6/16, 38%). Three apps targeted specific age groups (2 apps were for children aged 6-13 years and 1 app was for children aged ≥4 years). None of the apps provided tailored assessments or guidance for goal setting. None of the apps indicated that they were intended for the involvement of a health professional or had been tested for efficacy. The MARS total score indicated moderate app quality overall (mean 3.42, SD 0.49) and ranged from 2.5 to 4.2 out of 5 points. The Habitz app ranked highest on the MARS total score among HRB apps (score=4.2), whereas Thumsters ranked highest (score=3.9) among the WHRB apps. Subgroup analysis revealed a pattern of higher quality ratings in the HRB group than the WHRB group, including the mean MARS total score (mean 3.67, SD 0.34 vs mean 3.09, SD 0.46; *P*=.02); the engagement and information subscales; and the app-specific items about perceived impact on knowledge, attitudes, and behavior change.

**Conclusions:**

Several high-quality commercially available apps target parents to facilitate goal setting and tracking for child behavior change related to both health and nonhealth behaviors. However, the apps lack evidence of efficacy. Future research should address this gap, particularly targeting parents of young children, and consider individually tailored guided goal setting and involvement of health professionals.

## Introduction

### Background

Several taxonomies and reviews of health behavior change techniques (BCTs) indicate that goal setting is a foundational aspect of initiating and maintaining health behavior change [1-3]. Goal setting and tracking form the basis for conceptualizing, operationalizing, and documenting change and are intrinsic aspects of human behavior [4]. At the most basic level, the process of goal setting for individuals who have a desire to make behavioral changes includes the need to identify a goal, identify behaviors that need to be modified, generate specific strategies that will be used to implement the goal, a time frame for implementation of a plan, and a metric for how success will be defined [5,6]. Basic research in goal setting often integrates a framework from organizational behavioral theory for operationalizing key characteristics of the goal [5]. In health behavior research and practice, goal setting is often designed around development of SMART goals (specific, measurable, achievable, relevant, time-bound). Use of these common sense characteristics is commonly found in goal-setting interventions. However, regardless of the use of the SMART goals process, setting goals in general may optimize adherence to a process and the probability of goal achievement [7].

Behavior change research is not always able to provide specific guidance on the translation of theory into real-world activities to achieve health behavior change. For example, although goal-setting and tracking interventions may be informed by the social cognitive theory through the assessment of self-efficacy and facilitators and barriers related to success [8], the interaction of the individual with feedback, the use of peers in goal attainment, and engaging individuals in the change process may be implemented in many ways. Conceptually, self-efficacy may be the most studied construct related to goal attainment and has been related to the nature of the goals selected, the strength of commitment, and outcomes expectancies [8]. Other conceptual factors and processes that have been shown to correlate with goal implementation and achievement include individual abilities, use of feedback, goal commitment, relevant resources, level of stressors, and rewards for progress toward goal attainment [9]. However, these are factors that are characteristics of individuals and not part of the intervention itself. As such, any review of intervention or mobile app characteristics surrounding goal setting and achievement cannot predict or determine who will be successful in using the app regardless of the features.

After setting a goal, it is important to monitor progress to identify whether an individual is on track with regard to the nature of change, quantity of change, or timeline of the goal progress. Monitoring, or tracking, may also be used to document barriers to change or progress. The importance of this aspect of using goals in behavior change can be observed in the many health behavior tracking tools and apps available commercially. The importance of tracking behaviors is not only to raise awareness to the goal behavior on a regular basis through the act of tracking but also in the use of aggregated feedback over time. Patterns in goal achievement may be useful for identifying problems and refocusing attention or resetting a goal if it is not appropriate [10].

Goal setting and tracking are appealing for use by parents in relation to their children’s behavior because they provide a concrete process upon which to map and operationalize change, create opportunity for integrating adaptive assessment and personalized action plans, and may be used in a time-limited or cyclical manner. For example, tracking may be used to focus parental attention toward a goal on a daily or habitual basis. Nutrition and physical activity interventions that incorporate goal setting as a behavioral strategy have been generally evaluated as effective [8,11], including the incorporation of goal setting in childhood obesity prevention intervention studies focused on families, children, and adolescents [1,12,13]. Given the role of parents in creating the home food environment and making shopping choices, particularly for young children, the need for holistic family-based approaches to improve pediatric diet, weight, screen time, and physical activity has long been recognized [14,15].

Several recent studies have reviewed mobile health (mHealth) apps related to nutrition, physical activity, and weight management in children and adolescents. A 2015 review by Burrows et al [16] of 27 mobile apps for adolescents aged ≥13 years focused on weight management found that goal setting was the most common BCT, used in more than half of the apps (56%). A 2017 review by Schoeppe et al [17] assessed 25 mobile apps for child and adolescent users aged 2 to 18 years that focused on diet, physical activity, or sedentary behavior and found that one-third of the apps included goal setting as one of the BCTs. Finally, a 2022 review by Brown et al [18] of 259 nutrition-themed apps intended for children aged ≤12 years found that 18% of the apps included goal setting and planning as BCTs. In the review by Brown et al [18], approximately two-thirds of the child-focused apps were considered food games, and 17 of the apps were classified as “habit trackers,” which they defined as apps that enabled the children to log their food and drink intake. These reviews demonstrate interest in apps that support goal setting and tracking for child behavior, but they only included apps targeting children and adolescents as the app users, not apps that target parents as the primary users. In addition, although some of the reviewed apps included goal-setting features, this was not the focus of the reviews. Parents may wish to set goals to improve health-related behaviors (HRBs) and promote prosocial behaviors in their children, such as chores and homework.

Thousands of mHealth apps promote nutrition, physical activity, weight management, sleep, and other wellness behaviors for adults, but few are designed for parents to influence their children’s behaviors [19,20]. Little is known about the extent to which mobile apps that are designed to guide parents in using goal setting and tracking as BCTs for their children are commercially available or the intended context for apps to be used. For example, apps could be designed for parents to them at their home. Alternatively, apps could be used in coordination with health professionals in various contexts (eg, medical care, nutrition education, behavioral therapy, or personal coaching), such as sharing their app data with the health professional or using an app as part of a larger intervention [21]. Furthermore, little is known about the evidence base and quality of such existing apps [22,23].

### Objectives

Given the established evidence for goal setting and tracking as BCTs and the limited examination of mHealth apps in this domain in the context of families, we sought to address this gap. This study aims to review commercially available apps related to setting and tracking goals that target parents and children, both overall and specifically for apps that focus on HRBs. The specific objectives were to (1) classify the general characteristics, features, evidence base, and target behaviors of the apps and (2) assess the quality of the identified apps using an existing validated tool, overall and separately for the apps that target HRBs versus apps that do not include any HRBs. This review is a first step toward making recommendations for the use of mobile apps for goal setting and tracking with parents to influence child health behaviors.

## Methods

### Search Criteria and App Identification

The Apple App Store (iOS operating system) and Google Play (Android operating system) in the United States were searched once weekly between June and August 2021, using the following keywords to identify a pool of apps related to goal tracking for parents of children: *child goals*, *kids health goals*, *toddler parenting app*, *child nutrition goal tracking*, and *kids’ nutrition goals*. Relevant apps were also identified using a snowball approach through similar apps recommended by the app stores during searches.

Inclusion criteria included those apps with the primary purpose of assisting parents with goals related to their children’s behaviors, including setting goals, tracking goals, tracking goal progress or behaviors (good or bad), or giving feedback pertaining to goals. Only apps that were available for download in the United States were included. Exclusion criteria included apps that functioned primarily as scheduling or daily planners, list making, time management, allowance tracking, or finance management.

### Classification of Apps

App characteristics, features, and target behaviors were tracked and documented in an Excel (Microsoft Corp) sheet throughout the review process based on review of the app store description and app content. Certain app characteristics remained constant (eg, app name, developer, and Android or iOS availability), whereas others could change over time (ratings, downloads, reviews), so these were all documented on the same date (April 19, 2022). Features that were only available through paid upgrades were noted in the classification table. These paid upgrade features were reviewed based on descriptions and screenshots within the app and in the app store overview, and we did not directly test them.

All the app features were tabulated and described for each app. Then categories of features were created via the identification of commonly occurring themes. No app features were omitted from the assessment, and overlapping features were noted in the table. For each app, we documented the specific behaviors associated with possible goals that could be set and tracked. On the basis of the target behaviors, we assigned the apps to 1 of the 2 categories: apps with HRBs and the remaining apps without HRBs (WHRBs).

To characterize the evidence base, we reviewed the descriptions of the apps in the Apple App Store and Google Play, within the app, and on the developers’ websites, when applicable. In addition, we searched for the app names and developer names in PubMed, PsycINFO, and Google Scholar databases to locate any published research on the apps.

### Quality Evaluation

#### Use of the Mobile App Rating Scale

App quality was assessed using the Mobile App Rating Scale (MARS), a tool used to measure the quality of mobile apps. The MARS is a validated scale that is considered both objective and reliable [24]. The MARS has been used in similar app reviews and systematic app reviews pertaining to a variety of topics such as nutrition and physical activity for families and children [17,18,20,25,26]. The MARS quality subscale is composed of 19 items grouped into four domains: (1) engagement (5 items), (2) functionality (4 items), (3) aesthetics (3 items), and (4) information (7 items). Items are measured on a 5-point Likert scale, with 5 representing the highest quality. A score for each domain subscale is computed as the mean of the items in that domain. The total MARS quality score is computed as an average across the 4 domains and has demonstrated high internal consistency (Cronbach α=.90) [24]. The MARS instrument also includes a set of 6 app-specific items that can be used to assess the perceived impact of the app on users for the target behavior in terms of awareness, knowledge, attitudes, intention to change, help seeking, and the likelihood of actual change in the target health behavior (behavior change).

Each app was assessed independently by 2 reviewers using the MARS tool. The first author reviewed all the apps as the primary reviewer, and 4 additional authors served as the second reviewer on 4 apps each. All reviewers hold advanced degrees in their respective fields of nutrition, public health, psychology, sociology, or health informatics. All reviewers were trained using the web-based MARS training video and group discussion of the scoring instructions [27]. Reviewers were encouraged to refer back to the video, if necessary, to confirm their understanding of the most accurate and appropriate evaluation of each MARS domain. Differences greater than one point between the 2 reviewers were resolved by discussion and, if needed, a final decision by a third reviewer was applied when needed. Following the process of previous reviews using MARS [17], differences of one point in the assessments were resolved by taking the average of the 2 items.

#### Data Analysis

For the first objective, the general characteristics (ratings, installs, target age, etc), features (user options, available settings, visual displays, etc), evidence base, and health-related target behaviors for each app were tabulated and described. For the second objective, internal consistency of the total MARS quality scale and its subscales was calculated using Cronbach α. Descriptive statistics were used to summarize the mean and SD for the total MARS quality scale, 4 MARS quality subscales, and the 6 MARS app-specific items for each app individually, overall for all the apps combined, and separately for HRB apps and WHRB apps. As part of the subgroup analysis, we also tested for differences between the HRB apps and WHRB apps using a Kruskal-Wallis test, with a significance level of .05.

## Results

### App Identification

A total of 21 apps were downloaded during the keyword search and initial review of app descriptions in the app stores. Next, the downloaded apps were screened for the inclusion and exclusion criteria. Of these, 9% (2/21) of the apps did not meet the inclusion criteria for goal tracking (ie, KidBehave and Wello). Some apps used the word *habits*, not in line with the technical definition of habit from psychology but rather using the lay meaning that is equivalent to behaviors and goals [28], so these apps were retained. Furthermore, 9% (2/21) of the apps were excluded because they were allowance trackers (ie, Homey and Chores & Allowance Bot), and 1 app was excluded because it was not available for use in the United States (ie, Goalstar Rewards Notification). The remaining apps (n=16) were included in the review.

### Classification of Apps

General app characteristics and features of the 16 apps are presented in Tables 1 and 2. All apps were available on the Apple App Store (iOS) and 9 apps were available on Google Play (Android). The apps defined goals, tasks, and behaviors in different ways. For example, in 11 apps (ie, Habitz, FamJam, Points, Happy Kids Timer, Reward Chart, Go Hero, Smiles & Frowns, iReward, S’moresUp, Our Home, and Child Reward), goals were operationalized as trackable, desirable (or undesirable) behaviors, where points, stars, or some type of positive or negative feedback were associated with the behaviors. In such cases, the points, stars, or other tracking item could later be redeemed for a reward determined by the app or the user. Many of these apps often contained a predefined selection of goals, tasks, or behaviors (often under different categories) and the option for the user to add their own goals, tasks, or behaviors.

A total of 9 apps contained predefined goals that allowed users to track the following HRBs: nutrition and mealtime (n=6, 66%), physical activity and screen time (n=5, 55%), sleep (n=7, 77%), and personal hygiene (n=6, 66%; Table 1). Specific trackable behaviors that appear in each health-related category are listed in Table 3. Examples of trackable non–health-related behaviors include child routines, chores or home care, homework or school performance, attitudes or moods, and treatment of others. Moreover, 5 of the apps included in the WHRB category (ie, Thumsters, Stellar, punti, Points Wallet, and Chore Pad) did not include any predefined behaviors for goals and required the user to manually define their own behavior goals.

**Table 1:** Characteristics of commercially available family goal setting and tracking mobile apps for parents that target health-related behaviors (as of April 19, 2022).

				Apps targeting health-related behaviors (n=9)																
				iRewardChart		Points		OurHome		Happy Kids Timer		S’moresUp		Go Hero		FamJam		Smiles & Frowns		Habitz
**App characteristics**																				
	**Apple App Store (iOS)**																			
		Average rating (out of 5)	3.6		4.1		4.3		4.3		4.3		—^a^		4.6		4.2		4.4	
		Number of ratings	227		34		1500		750		697		—		29		43		642	
	**Google Play (Android)**																			
		Average rating (out of 5)	2.5		4.1		3.2		4.3		3.8		—		3.9		—		—	
		Number of ratings	669		217		>4000+		>19,000		783		—		90		—		—	
		Number of downloads	>10,000		>10,000		>500,000		>1,000,000		>100,000		>1,000		>10,000		—		—	
	Target child age range specified			—		—		—		—		4-6 years, 7-10 years, >11 years		—		—		—		6-13 years
	Tested in research studies or trials			No		No		No		No		No		No		No		No		No
**Features**																				
	Allows multiple children on family profile			P^b^		✓^c^		✓		—		✓		✓		✓		✓		✓
	Child user account available on same device			—		—		✓		—		✓		✓		✓		—		✓
	Family network option with children on separate devices			—		✓		—		—		✓		✓		—		✓		✓
	Option to choose cartoon icons for child			—		—		✓		—		✓		—		✓		—		✓
	Option to choose photo for child			✓		✓		—		—		✓		✓		✓		✓		—
	Provides individually tailored guidance on recommended goals			—		—		—		—		—		—		—		—		—
	Can set up multiple goals			✓, P^d^		✓		✓		✓		✓		✓		✓		✓		✓
	Allows parents to set rewards			✓		✓		✓		P		✓		✓		✓		✓		✓
	Can change reward values			—		✓		✓		—		✓		✓		✓		✓		—
	Visual chart for tracking over time			✓		✓		✓		✓		✓		✓		✓		✓		✓
	Additional features via paid upgrade (not reviewed)			—		—		—		P^e^		—		—		—		—		—
**Health-related target behaviors**																				
	Nutrition and mealtime			✓		—		✓		✓		✓		—		—		✓		✓
	Physical activity and screen time			✓		—		✓		—		✓		—		✓		—		✓
	Sleep			✓		✓		✓		✓		✓		✓		—		—		✓
	Personal hygiene			✓		—		—		✓		✓		—		✓		✓		✓

^a^Data not available in app store or the feature not included in the app.

^b^P: the feature available via paid upgrade (not directly tested in the review).

^c^✓: the feature included in the app and tested.

^d^Paid upgrade for >4 goals.

^e^Additional paid upgrades: custom order and time length of goals, email, or print reward certificate.

**Table 2:** Characteristics of commercially available family goal setting and tracking mobile apps for parents that target other behaviors (as of April 19, 2022).

				Apps targeting other behaviors (n=7)												
				Child Reward		Stellar		Chore Pad		Punti		Reward Charts		Points Wallet		Thumsters
**App characteristics**																
	**Apple App Store (iOS)**															
		Average rating (out of 5)	1.3		4.6		2.8		4.7		4.5		4.5		4.8	
		Number of ratings	4		276		24		6		13		53		717	
	**Google Play (Android)**															
		Average rating (out of 5)	3.3		—^a^		—		—		—		—		4.2	
		Number of ratings	405		—		—		—		—		—		203	
		Number of downloads	>50,000		—		—		—		—		—		>10,000	
	Target child age range specified		—		—		—		6-13 years		—		—		—	
	Tested in research studies or trials		No		No		No		No		No		No		No	
**Features**																
	Allows multiple children on family profile		✓^b^		✓		P^c^		✓		✓		✓		✓	
	Child user account available on same device		—		—		—		—		—		—		—	
	Family network option with children on separate devices		—		—		—		P		—		—		—	
	Option to choose cartoon icons for child		✓		—		—		—		—		—		—	
	Option to choose photo for child		✓		✓		✓		—		✓		✓		✓	
	Provides individually tailored guidance on recommended goals		—		—		—		—		—		—		—	
	Can set up multiple goals		✓		P		✓, P^d^		✓, P^e^		✓		—		✓	
	Allows parents to set rewards		✓		—		—		—		✓		✓		—	
	Can change reward values		✓		—		✓		—		—		✓		✓	
	Visual chart for tracking over time		✓		—		—		✓		✓		—		✓	
	Additional features via paid upgrade (not reviewed)		—		—		—		P^f^		—		—		—	

^a^Data not available in app store or the feature not included in the app.

^b^✓: the feature included in the app and tested.

^c^P: the feature available via paid upgrade (not directly tested in the review).

^d^Paid upgrade for >4 goals.

^e^Paid upgrade for >5 goals.

^f^Daily reminders to track goals.

**Table 3:** Apps containing features that allow users to track health-related goals.

Name of app			Description of features
**Apps that track nutrition, food, or mealtime-related behaviors (n=6)**			
	Habitz	Eat healthier foods (12 types of food choice goals may be selected); cut out unhealthy foods (5 types of food choice goals may be selected)	
	Happy Kids Timer	Can select the activities linked to the goal of completing a morning or nighttime routine: eat your breakfast, pack your lunch	
	iRewardChart	No junk food, sit through the meal, eat fruit	
	OurHome	Kitchen, meals, shopping	
	S’moresUp	Cook simple foods, set and clear table, no device at mealtime, cook simple meal with supervision, drink water and stay hydrated, eat breakfast, help make and pack lunch, learn to read labels on food, make breakfast, make your own snacks, help make dinner, learn to read labels on food	
	Smiles & Frowns	Using good table manners, refusing to eat properly (negative), behaving poorly at the table (negative)	
**Apps that track physical activity and screen time behaviors (n=5)**			
	FamJam	Exercise	
	Habitz	Physical activity, 2 hours screen time	
	iRewardChart	Exercise	
	OurHome	Exercise	
	S’moresUp	Device limitations (homework first, during family time, at school, etc), play outside for 45 min, practice sports for 45 min	
**Apps that track sleep, sleep routine, or bedtime behaviors (n=7)**			
	Go Hero	Evening ritual	
	Habitz	On time bedtime last night, Woke up on time	
	Happy Kids Timer	Can select and complete a series of activities linked to the goal of completing a nighttime routine and going to sleep (eg, toilet, pajamas, read a book, lights off, go to sleep)	
	iRewardChart	On time to bed	
	OurHome	Bedroom, bedtime routine	
	Points	Bedtime on time	
	S’moresUp	No screen within an hour of bedtime, sleep at least 10 hours per day, wake up on time, get ready for bed	
**Apps that track personal hygiene–related behaviors (n=6)**			
	FamJam	Brush teeth, washing hands	
	Habitz	Brush your teeth	
	Happy Kids Timer	Can select the activities linked to the goal of completing a morning or nighttime routine: brush your teeth and wash your hands, take a shower or bath	
	iRewardChart	Brush teeth, take bath	
	Smiles & Frowns	Brushing teeth, washing up or bathing, not washing or brushing teeth (negative)	
	S’moresUp	Deodorant, brush teeth, take a shower or bath, wash hands	

Among the 9 HRB apps in the Apple App Store, OurHome had the highest number of user ratings (>1500 ratings), followed by Happy Kids Timer (750 ratings), S’moresUp (697 ratings), and Habitz (642 ratings; Table 1). The highest average user ratings were obtained for FamJam (4.6 rating) and Habitz (4.4 rating). The Apple App Store does not report the number of downloads. Among the 7 HRB apps on Google Play, Happy Kids Timer had the highest number of user ratings (>19,000 ratings), highest average rating (4.3 rating), and highest number of downloads (>1 million). OurHome had the second highest number of user ratings (>4000 ratings) and downloads (>500,000 downloads), but Points had the second highest average user rating (4.1 rating).

Among the 7 WHRB apps in the Apple App Store, the apps with the highest user ratings were Thumsters (717 ratings) and Stellar (276 ratings; Table 2). Thumsters also had the highest average user rating (4.8 rating), followed by punti (4.7 rating) and Stellar (4.6 rating). Among the 2 WHRB apps on Google Play, Child Reward had the largest number of downloads (>50,000 downloads) and user ratings (405 ratings), whereas Thumsters had the highest average user rating (4.2 rating).

Overall, 3 apps explicitly stated that the apps were designed for children of specific age ranges. One HRB app (Habitz) and one WHRB app (Punti) were targeted for children aged 6 to 13 years. Another HRB app (S’moresUp) offered a feature to select the age range of children to tailor some of the visuals and features for 3 separate age groups (4-6 years, 7-10 years, and >11 years).

Furthermore, 5 apps offered optional features through paid upgrades, which were described in the apps and app stores. Of these 5 apps, 4 (80%) apps (iRewardChart, Stellar, Chore Pad, and punti) included the paid option to remove limits on 2 existing features—the number of child profiles and the number of goals. The paid upgrade options in the fifth app, Happy Kids Timer, allowed the parents to specify rewards, to customize the order and time length of goals, and to email or print reward certificates. In addition, punti offered paid upgrade options to sync multiple devices on a family network and to provide daily reminders about tracking goals.

In a review of the app store descriptions, information within the app, and developer websites, research was only mentioned on the websites for 2 apps, Habitz and S’moresUp, which stated that research was consulted to inform the development of these apps. The Habitz website described that the development team included experts in nutrition, psychology, and pediatric behavior, and it also mentioned that the team had conducted internal testing of the app, but no details were provided. Searches of scientific databases did not reveal any publications indicating that the apps had been tested in published clinical trials or other studies.

### Quality Evaluation

The MARS quality rating tool demonstrated high internal consistency and reliability for rating the goal-setting and tracking apps, with a Cronbach α of .92 for the total score, and high scores for the 4 subscales: engagement (α=.80), functionality (α=.85), aesthetics (α=.88), and information (α=.82).

The MARS app quality ratings for each reviewed app and overall for the 16 apps are reported in Table S1 in Multimedia Appendix 1. Overall, the apps had a mean total score of 3.4 (SD 0.5). Among the HRB apps, Habitz had the highest score of 4.2, followed by Smiles & Frowns (score 4.1) and FamJam and Go Hero tied for third (score 3.8). In addition, Habitz had the highest subscale scores for engagement, aesthetics, and information, whereas Smiles & Frowns had the highest score for the functionality subscale. For the top-ranked WHRB apps, Thumsters ranked first (score 3.9), followed by Reward Chart (score 3.2), Points Wallet (score 3.2) and punti (score 3.2) tied for the second. Thumsters had the highest subscale scores for engagement, functionality, and aesthetics, and it tied with Reward Chart and Points Wallet for the highest score on the information subscale score.

With regard to the lowest ranked HRB apps, iRewardsChart and Points ranked lowest in total score and ranked low in most of the subscales, except for functionality. Comparably, among the WHRB apps, Child Reward and Stellar ranked the lowest overall, with Child Reward receiving a score of 1.9 in the area of information and Stellar receiving a score of 1.9 for the engagement subscale. With regard to the 6 goal-setting scores, we observed that Child Reward and Stellar had the lowest scores, with Stellar receiving the lowest scores of all ranked categories, with a consistent 1.5 score in 5 out of the 6 goal-setting categories.

Table S1 in Multimedia Appendix 1 lists the scores for the 6 app-specific items that reflected reviewers’ perceived impact of the apps on aspects of goal setting and goal tracking. Among the HRB apps, 4 apps (Habitz, Smiles & Frowns, FamJam, and Go Hero) received ≥4.0 in at least 4 of the 6 items. Smiles & Frowns had the most items scored at 4.5, followed by Habitz and Go Hero. At the same time, Habitz was ranked first in the important category of behavior change, and 4 other apps tied for the second highest score in behavior change (score 4.0).

Among the WHRB apps, Thumsters received the highest ratings on the app-specific items, with a score of 4.5 on 4 of the items. Reward Chart had the next highest ratings, with a score of ≥3.5 on 4 items. Thumsters scored the highest on the behavior change item (score 4.0), followed by Chore Pad (score 3.5).

Figure S1 in Multimedia Appendix 1 illustrates the ranking of the total score for each app, with the HRB apps in black and the WHRB apps in gray. Notably, 8 of the top 9 ranked apps were HRB apps. The exception was Thumsters, which ranked third overall and received the highest total score among the WHRB apps.

This pattern was consistent in the mean comparison tests of the MARS quality scores for the HRB apps and the WHRB apps, as presented in Table 4. We observed a significantly higher total score for the HRB apps (mean 3.67, SD 0.34) compared with the WHRB apps (mean 3.09, SD 0.46; *P*=.02). Among the subscales, the engagement subscale was higher for the HRB apps (mean 3.60, SD 0.30) compared with the WHRB apps (mean 2.86, SD 0.60; *P*=.01), and the information subscale was higher for the HRB apps (mean 3.03, SD 0.44) compared with the WHRB apps (mean 2.86, SD 0.3; *P*=.01). The functionality and aesthetics subscales did not differ significantly between the 2 groups.

Among the app-specific items on reviewers’ perceived impact of the apps on goal setting and goal tracking, we observed that three of the six items were significantly higher for HRB apps versus WHRB apps: (1) knowledge (HRB apps: mean 3.83, SD 0.43; WHRB apps: mean 3.00, SD 0.91; *P*=.02), (2) attitudes (HRB apps: mean 3.83, SD 0.35; WHRB apps: mean 2.86, SD 0.9; *P*=.01), and (3) behavior change (HRB apps: mean 3.78, SD 0.44; WHRB apps: mean 3.07, SD 0.53; *P*=.02). The 2 groups of apps did not differ significantly in the perceived impact of the apps on awareness, intention to change, or help seeking.

**Table 4:** Comparison of Mobile App Rating Scale scores for apps targeting health-related behaviors versus other apps.

					Apps targeting health-related behaviors (n=9), mean (SD)			Apps targeting other behaviors (n=7), mean (SD)			*P* value
**App quality**											
	Total score			3.67 (0.34)			3.09 (0.46)			.02	
	**Subscales**										
		Engagement	3.60 (0.3)			2.86 (0.6)			.01		
		Functionality	4.04 (0.61)			3.70 (0.61)			.18		
		Esthetics	4.02 (0.69)			3.33 (0.54)			.08		
		Information	3.03 (0.44)			2.48 (0.3)			.01		
**App-specific items (perceived impact on goal setting or tracking)**											
	Awareness			3.83 (0.66)			3.21 (0.95)			.13	
	Knowledge			3.83 (0.43)			3.00 (0.91)			.02	
	Attitudes			3.83 (0.35)			2.86 (0.90)			.01	
	Intention to change			3.83 (0.35)			2.93 (1.02)			.07	
	Help seeking			3.28 (0.44)			2.93 (0.73)			.37	
	Behavior change			3.78 (0.44)			3.07 (0.53)			.02	

## Discussion

### Principal Findings

In summary, more than half of the 16 goal-setting and tracking apps in this review targeted HRBs, which included behaviors related to nutrition and mealtime, physical activity and screentime, sleep, and personal hygiene. Only 3 apps were tailored for specific ages of children, including 2 apps for middle childhood to early adolescence, and 1 app for 3 age groups spanning from 4 years through adolescence. No evidence was found indicating that any of the apps had been tested in clinical trials, experimental studies, or other studies. Most of the apps offer features for personalizing the user experience of tracking multiple children, and some of the apps offer the option for both parents and children to directly use the apps together as a family. However, none of the apps provided individually tailored assessments or guidance to recommend specific goals for each child, and none suggested using the app in coordination with their children’s health care provider.

The app quality assessment indicated an overall moderate app quality and substantial variation in quality ratings, with only a handful of apps consistently scoring high across subscales and app-specific items. Among the HRB apps, Habitz had the highest MARS quality rating and the third-highest user rating in the Apple App Store, and among the WHRB apps, Thumsters ranked the highest on both. Aside from these 2 apps, the rank order of apps in our MARS quality ratings and in the average user ratings from the app stores were fairly inconsistent. Ranking the apps and descriptive subgroup analysis revealed a pattern of higher quality ratings in the HRB group than the WHRB group, including the MARS total score, the engagement and information subscales, and the app-specific items about perceived impact on knowledge, attitudes, and behavior change.

### Comparison With Prior Work

To our knowledge, this is the first review of commercially available mobile apps that specifically target parents and include goal-setting features. Three recent reviews of commercially available mobile apps targeting HRBs are relevant for comparison with our study because the authors reported that a substantial proportion of the reviewed apps included goal-setting BCTs, ranging from 18.1% (47/259, including 17 “habit trackers”) [18] to 32% (8/25) [17] to 56% (15/27) [16]. The key difference is that these reviews focused on apps that targeted children and adolescents as the primary users rather than parents. Although 7 of the 9 HRB apps in our review provided the option for children to directly use the apps in addition to the parents, none of the apps included in those reviews overlapped with the apps reviewed in our study. Possible reasons for the lack of overlap are that the authors reviewed apps that were available on the app stores in their respective countries and in different years—ours in the United States in 2021 versus the others in Australia in 2013 [16] and in 2016 [17] and Canada in 2018 to 2019 [18].

Two of these reviews assessed app quality using the MARS tool [17,18]. Schoeppe et al [17] reported a mean MARS total score of 3.6 out of 5 possible points for the 25 nutrition and physical activity–focused apps (range 2.4-4.4), indicating moderate quality, but the subgroup of 8 apps with goal-related BCTs had a higher mean of 4.0 and less variation (range 3.6-4.4). Brown et al [18] indicated that the mean MARS total score for the 259 nutrition-related apps was also 3.6 (range 2.2-4.7), or moderate quality, and indicated a similar mean of 3.5 with less variation (range 3.2-4.2) for the subgroup of 17 apps in the “habit tracker” subgroup. In comparison, in this review, all apps included goal setting or tracking, with a mean MARS total score of 3.4 (range 2.5-4.2), and the subgroup of HRB apps, most of which included nutrition or physical activity, had a slightly higher mean of 3.7 (range 3.2-4.2). Thus, the quality scores for the HRB subgroup in our study fell in between the relevant subgroups in the reviews by Brown et al [18] and Schoeppe et al [17].

The highest scoring MARS subscale was functionality for the review by Schoeppe et al [17] (4.10 overall; 4.3 goal subgroup), Brown et al [18] (4.0 overall; 4.0 habit tracker subgroup), and our review (3.9 overall; 4.0 HRB subgroup). However, the reviews differed in the lowest-scoring MARS subscale. Engagement was lowest for the review by Brown et al [18] (2.9 overall; 3.3 habit tracker subgroup), whereas information was lowest for the review by Schoeppe et al [17] (2.8 overall; 3.2 goal subgroup) and our study (2.8 overall; 3.0 HRB subgroup). These findings across reviews suggest that commercial apps for parents and children that focus on HRBs or use goal setting as a BCT tend to perform well on the functionality domain of MARS, which includes performance, ease of use, navigation, and gestural design, but they may be weaker on the information and engagement domains.

In the third review, Burrows et al [16] noted that none of the 27 apps related to child weight management that were commercially available in 2013 were tested through research studies, which was also found in our review. They also noted that most of the app developers did not report on the involvement of content area experts, which was the same for all but one of the apps in our review. Furthermore, most of the apps in the review by Burrows et al [16] contained content that they deemed was not consistent with relevant best practices, that is, national dietary guidelines. Although the review by Burrows et al [16] did not include a quantitative quality assessment using a tool, such as MARS, the findings are consistent with our finding related to the information domain of MARS. The items comprising this domain include clear and achievable goals, correct and comprehensive information, credibility of the app source or developer, and the scientific evidence base of the app [24]. In our review, credibility and evidence base were consistently the lowest-scoring items, which reduced information domain scores. This finding of a low information domain score driven by low ratings on the credibility and evidence base items is also consistent with reviews of commercially available apps for adults that focus on nutrition, physical activity, and other health promotion behaviors [20,29].

Although the HRB apps with goal-setting features reviewed here were not tested through research, the specific HRBs that the apps targeted through predefined goal options were largely consistent with the current scientific literature and best practices related to child and adolescent health. For example, 3 of the 6 apps that target nutrition and mealtime behaviors had predefined goal options on child consumption of specific healthy and unhealthy food options, such as increasing intake of vegetables, fruit, or water, and decreasing intake of junk food (eg, fast food and sugar-sweetened beverages). Promoting goals targeting these behaviors has the potential to positively influence child dietary patterns based on the existing scientific evidence related to these broad behaviors [23,30-35]. Similarly, 4 apps offered predefined goal options for increasing physical activity (eg, general exercise, outdoor play, and practicing sports) and 2 apps included goal options for limiting screen time, reducing total screen time, and contingent access to electronic devices. Both increased physical activity and decreased screen time have been consistently linked to health and other benefits for children [36-40]. Moreover, 7 of the apps included sleep and bedtime routine behaviors for goals. Both sleep duration and sleep quality have been associated with a number of health-related outcomes in children, such as physical development, cognitive development, emotional and behavioral problems, and excessive weight gain [41-44]. Furthermore, 6 of the apps included predefined goals related to personal hygiene in children, such as brushing teeth, washing hands, and bathing, which support current oral health guidelines [45,46], effective public health hand hygiene strategies [47-49], and other benefits for young children related to sleep and emotional connection with caregivers [50]. Therefore, although the parent-targeted apps we reviewed have not been individually tested for efficacy, our findings suggest that some of the apps focus on HRBs that have a general scientific evidence base and could potentially be leveraged for health promotion and chronic disease prevention.

Interestingly, our review identified a smaller number of commercially available parent-targeted apps than the number of child-targeted apps identified in previous reviews, even after restricting to apps that include goal-related features [18]. The large number of mobile apps targeting young children as the primary users is inconsistent with the vast scientific literature that has established the crucial role of parental influence and environmental changes in shaping children’s behavior before adolescence [14,15,51]. This notion is supported by the concept of parental health-related empowerment by Gago et al [15], which they define as “the process by which parents realize control over their life situation and take action to promote a healthier lifestyle.” Their work demonstrated that increasing parental empowerment can improve weight-related parenting practices, which in turn can influence children’s behaviors and promote prevention of obesity and chronic diseases.

### Limitations

Although this is one of the only objective reviews of parent-targeted goal-setting apps for child-focused behavior change, we acknowledge its limitations. Although a strength of the evaluation process was the use of a validated instrument that has been successfully used in many previous studies and provides valuable insights, the evaluation did not include using the apps with the target population of parents. It is possible that parents would have rated the quality domains differently, thus limiting the inferences we can make regarding the utility and engagement features. Although the inclusion of actual use-case testing was not feasible and outside of the scope of the review, this can be pursued in future research.

Second, because of the dynamic nature of the app stores that do not indicate the number of apps meeting search criteria, it is possible that eligible apps could have been missed. We mitigated this challenge by using multiple search terms and by using a snowball approach of reviewing similar apps recommended by the app store when viewing each app that appeared in the searches. Finally, we did not directly test features that were only available through paid upgrades to maintain consistency across the apps and because paid versions are likely not accessible for all parents. It is possible that some of the MARS ratings, particularly the app-specific items, may have scored higher when including the paid upgrades.

### Implications for Research and Practice

The overall average app quality was rated as moderate in this review of commercially available apps that target parents to facilitate goal setting and tracking for child behavior change. Of note, several of the apps were rated as high quality, particularly among the apps that target HRBs, and several were rated as having strong potential to generate changes in knowledge, attitudes, and behaviors. However, even the apps with high-quality ratings lack documented evidence of efficacy. Although the field of mHealth continues to grow along with technological advances, potential mHealth adaptations of existing behavioral intervention strategies may or may not offer improvement over traditional delivery formats; for example, Is goal setting more effective for parents when they are prompted during a conversation with their child’s physician or when they are prompted by a mobile app while they are at home [52]? Furthermore, the context in which parents interact with mobile apps related to goal setting and tracking will likely influence the effectiveness on behavior initiation and maintenance. For example, a parent could set a goal to initiate a new food purchasing behavior during a visit with a nutritionist at a public assistance program, then use a mobile app provided by the nutritionist when they go home to track goal progress, receive ongoing reinforcement, and share their progress with the nutritionist [53-55].

The provision of evidence supporting the impact of the apps is relevant for parents, clinicians, and other health professionals and could positively influence parent uptake if an app was supported scientifically. An evidence-based rating system could guide health professionals in identifying the most effective apps to recommend to parents of children in clinical settings and public health programs [56]. This is critical given that behavior change can be a challenging process and may require repeated attempts to be successful. Although the apps reviewed here may be efficacious, health professionals and parents currently have no basis for recommending or selecting an app for their families. In addition, apps that have not been proven effective could waste parents’ time and energy on strategies that are not likely to improve the target behavior. This represents a missed opportunity to engage parents in evidence-based strategies, and in some cases, it could potentially pose an ethical dilemma if health care professionals recommend ineffective apps to parents. Future app development and research should focus on this opportunity.

More research is needed to assess whether using a mobile app for parents to set and track goals for child HRBs is effective using existing apps or newly developed apps. Furthermore, future research should examine which specific goals or combination of goals within each behavior domain are most important, how they interact with each other, and the mechanisms that lead to changes in health outcomes [57]. For example, while it has been established that certain aspects of children’s eating routines such as family member presence, frequency of family meals, frequency of fast food consumption, positive mealtime atmosphere, parental modeling, and longer meal duration are associated with diet quality and health outcomes [58-61], it is difficult to establish the mechanisms that lead to weight-related outcomes of these routines and practices. Furthermore, the dietary and weight-related goals of motivated parents trying to establish positive mealtime routines and climates can be easily thwarted by factors such as strong child food preferences, preservation of the child’s self-esteem, desire for conflict avoidance, and inflexible time and finances [62].

Although parents were the primary users of the apps in this review, most of the HRB apps also offered options for the children to engage with the app, either as another user on the parent’s phone or on the child’s own device, linked together as a family. These features offer the opportunity to include the whole family in change, which could potentially enhance the impact of the apps as family-based interventions. A small but growing body of research is beginning to explore the delivery of family-based interventions through mobile apps, which show promise, but much more research needs to be done [63-65]. This could also provide an opportunity for parents to connect with the children’s health care provider to share the family’s goal progress data for remote monitoring and feedback. An important dimension to explore is developmentally appropriate levels of involvement for children of different ages, potentially with more involvement as children age. A notable gap identified in this review was the lack of goal-setting apps for parents of young children, with only one targeting parents of children aged 4 to 6 years and none targeting parents of children aged <4 years. During the infant, toddler, and preschool years, parents obviously exert a great influence on their children and have great potential to initiate healthy behaviors even at those young ages.

Finally, another gap identified in this review was that none of the apps provided validated assessments to guide goal selection or other individually tailored guidance to help recommend goals for children based on their unique needs. Previous research has demonstrated the feasibility and effectiveness of guided goal setting in in-person intervention settings [66-68], and more research is needed to extend and evaluate this strategy using mHealth and mobile technology in general.

### Conclusions

This review identified several high-quality commercially available apps that target parents with a focus on goal setting and tracking for child behavior change, both for HRBs and for non–health-related behaviors. However, these apps lack documentation of research-tested effectiveness. Future research should address this gap in the literature. In particular, more research is needed to assess the effectiveness of leveraging mobile apps to facilitate individually tailored guided goal setting for parents of young children. In addition, future research should explore options for integrating parent-focused apps as a complement to and reinforcement of health professionals’ interactions with parents around goal setting and tracking.

## Appendix

Multimedia Appendix 1 Supplemental Table 1. Mobile App Rating Scale scores for each reviewed app and overall (N=16). Supplemental Figure 1. MARS total quality score for reviewed apps.

## Abbreviations

BCT: behavior change technique
HRB: health-related behavior
MARS: Mobile App Rating Scale
mHealth: mobile health
SMART: specific, measurable, achievable, relevant, time-bound
WHRB: without health-related behavior
