# Supplementary material for: Commercially Available Mobile Apps With Family Behavioral Goal Setting and Tracking for Parents: Review and Quality Evaluation
Source: JMIR Pediatr Parent. 2023 Oct 13;6:e41779. doi: 10.2196/41779 (PMC10612003; doi:10.2196/41779)
Supplement: Multimedia Appendix 1 [file pediatrics_v6i1e41779_app1.docx]

**Appendix 1: Supplemental Table and Figure**

**Supplemental Table 1.** Mobile App Rating Scale scores for each reviewed app and overall (N=16).

|  | | | Apps targeting health-related behaviors (n=9) | | | | | | | | | Apps targeting other behaviors (n=7) | | | | | | | All, mean (SD) |
| --- | --- | --- | --- | --- | --- | --- | --- | --- | --- | --- | --- | --- | --- | --- | --- | --- | --- | --- | --- |
|  | | | iRewardChart | Points | OurHome | Happy Kids Timer | S'moresUp | Go Hero | FamJam | Smiles & Frowns | Habitz | Child Reward | Stellar | Chore Pad | punti | Points Wallet | Reward Chart | Thumsters |  |
| **App quality** | | | | | | | | | | | | | | | | | | | |
|  | Total score | | 3.2 | 3.3 | 3.5 | 3.5 | 3.6 | 3.8 | 3.8 | 4.1 | 4.2 | 2.5 | 2.7 | 3.0 | 3.2 | 3.2 | 3.2 | 3.9 | 3.4 (0.5) |
|  | **Subscales** | | | | | | | | | | | | | | | | | | |
|  |  | Engagement | 3.1 | 3.4 | 3.3 | 3.5 | 3.8 | 3.9 | 3.8 | 3.6 | 4.0 | 2.5 | 1.9 | 3.1 | 2.7 | 3.2 | 2.8 | 3.8 | 3.3 (0.6) |
|  |  | Functionality | 4.0 | 4.4 | 3.9 | 4.4 | 2.8 | 4.0 | 3.8 | 5.0 | 4.2 | 2.9 | 3.4 | 3.2 | 4.0 | 3.8 | 3.9 | 4.8 | 3.9 (0.6) |
|  |  | Esthetics | 3.2 | 2.8 | 4.2 | 3.5 | 4.5 | 4.3 | 4.7 | 4.2 | 4.8 | 2.5 | 3.2 | 3.2 | 3.3 | 3.3 | 3.5 | 4.3 | 3.7 (0.7) |
|  |  | Information | 2.5 | 2.5 | 2.8 | 2.8 | 3.3 | 3.0 | 3.1 | 3.5 | 3.8 | 1.9 | 2.3 | 2.4 | 3.2 | 2.7 | 2.7 | 2.7 | 2.8 (0.5) |
| **App-specific items (reviewers’ perceived impact on goal setting or tracking)** | | | | | | | | | | | | | | | | | | | |
|  | Awareness | | 4.0 | 3.5 | 3.5 | 4.0 | 2.5 | 4.5 | 3.5 | 4.5 | 4.5 | 2.5 | 1.5 | 3.5 | 3.5 | 3.5 | 3.5 | 4.5 | 3.6 (0.8) |
|  | Knowledge | | 3.5 | 3.5 | 3.5 | 3.5 | 3.5 | 4.5 | 4.0 | 4.5 | 4.0 | 2.5 | 1.5 | 3.0 | 3.0 | 3.0 | 3.5 | 4.5 | 3.5 (0.8) |
|  | Attitudes | | 4.0 | 3.5 | 3.5 | 3.5 | 3.5 | 4.0 | 4.0 | 4.5 | 4.0 | 2.5 | 1.5 | 3.0 | 2.5 | 3.0 | 3.0 | 4.5 | 3.4 (0.8) |
|  | Intention to change | | 3.5 | 3.5 | 3.5 | 4.0 | 3.5 | 4.0 | 4.0 | 4.5 | 4.0 | 2.5 | 1.5 | 3.0 | 2.5 | 2.5 | 4.0 | 4.5 | 3.4 (0.8) |
|  | Help seeking | | 3.5 | 3.0 | 3.5 | 2.5 | 3.5 | 4.0 | 3.0 | 3.0 | 3.5 | 2.5 | 1.5 | 3.5 | 3.0 | 3.0 | 3.5 | 3.5 | 3.1 (0.6) |
|  | Behavior change | | 4.0 | 3.0 | 3.5 | 3.5 | 4.0 | 3.5 | 4.0 | 4.0 | 4.5 | 2.5 | 2.5 | 3.5 | 3.0 | 3.0 | 3.0 | 4.0 | 3.5 (0.6) |

**Supplemental Figure 1.** MARS total quality score for reviewed apps

Health-related behaviors

Without health-related behaviors
